# Multiple cross displacement amplification (MCDA) for rapid detection of toxigenic *Clostridioides difficile* as a potential point-of-care testing

**DOI:** 10.1128/spectrum.03030-24

**Published:** 2025-07-22

**Authors:** Hui Hu, Xiaomei Zhang, Mengting Cai, Xiaojun Song, Hongrui Cheng, Fangya Zhai, Yulei Tai, Zheng Xu, Shan Lin, Yu Chen, Yun Luo, Dazhi Jin

**Affiliations:** 1 School of Laboratory Medicine, Zhejiang Provincial People’s Hospital (Affiliated People’s Hospital), Hangzhou Medical College 680617, Hangzhou, Zhejiang, China; 2 Key Laboratory of Biomarkers and In Vitro Diagnosis Translation of Zhejiang Province, Hangzhou, Zhejiang, China; 3 School of Biotechnology and Biomolecular Sciences, University of New South Wales 7800, Sydney, Australia; 4 Diago Biotech Pty Ltd, Sydney, New South Wales, Australia; London Health Sciences Centre, London, Ontario, Canada

**Keywords:** toxigenic *Clostridioides difficile*, MCDA, *tcdB* gene, point-of-care testing

## Abstract

Toxigenic *Clostridioides difficile* is the cause of *C. difficile* infection, highlighting the critical need for rapid and easy-to-use detection. In this study, a multiple cross displacement amplification (MCDA) assay was developed to detect toxigenic *C. difficile* and evaluated against real-time PCR and VIDAS *C. difficile* toxin A & B assay (CDAB). The results showed that MCDA targeting the *C. difficile tcdB* gene had a limit of detection (LOD) of 12.5 fg (2.5 copies of genomic DNA) per reaction with no non-specific amplification from other pathogens. The LOD for MCDA was the same as real-time PCR but had significantly faster detection time, detecting 500 pg and 12.5 fg per reaction in 5.7 ± 0.5 and 21.35 ± 2.2 min, respectively. Among 201 stool samples, MCDA identified 40 positive for toxigenic *C. difficile* with a shorter average detection time of 16.08 ± 4.62 min compared to real-time PCR (39.82 ± 4.44 min). Using cell cytotoxicity neutralization assays as reference, MCDA demonstrated a significantly higher specificity (92.4%) and a positive predictive value (67.5%) than real-time PCR (80.8 and 43.2%, respectively; *P* < 0.05). MCDA also had a significantly higher sensitivity (93.1%) and a negative predictive value (98.8%) compared to VIDAS CDAB (34.5 and 90.1%, respectively; *P* ≤ 0.001). This was also higher compared to real-time PCR (86.2 and 97.2%, *P* > 0.05). MCDA offers a promising screening alternative for point-of-care testing to detect toxigenic *C. difficile* and effectively rule out non-toxigenic *C. difficile*. It is rapid, easy to use, and has a balanced performance, making it applicable in clinical and community settings, particularly in resource-limited settings.

**IMPORTANCE:**

Rapid detection of toxigenic *Clostridioides difficile* is important for early intervention and outbreak control of *C. difficile* infection in primary healthcare facilities. This study developed an MCDA assay and showed that it was rapid, sensitive, user-friendly, and a suitable point-of-care screening alternative for accurately detecting toxigenic *C. difficile*. MCDA had the same sensitivity as real-time PCR while providing significantly faster turnaround times and improved specificity along with positive and negative predictive values. Its balanced performance and rapid detection capability make it well-suited for clinical detection of toxigenic *C. difficile* and point-of-care testing in communities, particularly in resource-limited settings, and for potential self-testing.

## INTRODUCTION

*Clostridioides* (*Clostridium*) *difficile* is a major cause of antibiotic-associated diarrhea worldwide (1). *C. difficile* infection (CDI) leads to various clinical manifestations, ranging from asymptomatic carriage to severe, life-threatening complications, such as sepsis, toxic megacolon, and pseudomembranous colitis. These complications are significant contributors to its high morbidity and mortality (2, 3). Over the past two decades, *C. difficile* has emerged as one of the most critical agents causing nosocomial enteric infections (4, 5). While hospital-associated CDI cases declined from 2011 to 2017, community-acquired CDI (CA-CDI) cases have risen (6). In the United States, CA-CDI accounts for approximately half of the nearly 223,900 infections and 12,800 deaths in 2017 (6, 7). Therefore, the timely identification of toxigenic *C. difficile* and the development of rapid, point-of-care detection tools are crucial for effective CDI diagnosis and for the prevention of the transmission of toxigenic *C. difficile* (8).

Numerous methods are available for the laboratory diagnosis of CDI based on the detection of toxigenic *C. difficile* (9). The well-accepted standards are toxigenic culture and cell cytotoxicity neutralization assay (CCNA); however, these approaches are labor-intensive, time-consuming, and subjective, limiting their use in clinical settings (10). Routinely used laboratory methods include enzyme immunoassay (EIA) and nucleic acid amplification test (11–13). EIAs exhibit variable sensitivities ranging from 60 to 85% (14) and are usually influenced by factors, such as patient age, immune status, and disease course, potentially leading to false negatives and delayed treatment (15). Molecular techniques, such as PCR-based assays targeting the *C. difficile tcdB* gene, offer superior sensitivity and specificity, provided that appropriate stool specimens are obtained by clinical laboratories. However, PCR typically requires standard laboratories and specialized instruments (16). Although PCR-based assays cannot accurately distinguish CDI from asymptomatic *C. difficile* colonization (CDC) (14), high negative predictive values allow them to effectively exclude patients with non-toxigenic *C. difficile* (17). Thus, the Infectious Diseases Society of America and the Society for Healthcare Epidemiology of America (IDSA/SHEA) guidelines support the use of molecular testing alone for early diagnosis in high-risk patients (defined as ≥3 unexplained diarrheal episodes within 24 h and recent antibiotic exposure) and when institutions have pre-agreed criteria for stool sample submission (18). Nevertheless, the requirement for specialized personnel and equipment limits the applicability of PCR-based assays in point-of-care settings (16). Currently, there is no single assay with high sensitivity and specificity, short turnaround time, and low cost for CDI diagnosis (17).

To date, isothermal nucleic acid amplification techniques have been applied for the detection of toxigenic *C. difficile* in stool samples, showing a promising performance (19). Compared to PCR and its derivatives, which require high temperatures and thermal cycling for primer annealing, isothermal amplification techniques operate at a constant temperature and do not require thermal cyclers, making them more suitable for rapid, on-site detection (20). Multiple cross displacement amplification (MCDA), an isothermal nucleic acid amplification technique developed in 2015, has emerged as a promising alternative for the detection of pathogens (21). MCDA uses a set of 10 primers (displacement primers F1 and F2, cross primers CP1 and CP2, and amplification primers C1, C2, D1, D2, R1, and R2) to enhance specificity and facilitate rapid detection, addressing limitations found in PCR and other isothermal nucleic acid amplification approaches (16). MCDA has also shown a promising performance for detecting various pathogens, including *Salmonella*, *Shigella*, *Listeria monocytogenes*, *Escherichia coli*, and severe acute respiratory syndrome coronavirus 2 (SARS-CoV-2), highlighting its potential as a prospective point-of-care testing option for pathogen detection (21–23).

In this study, we developed and evaluated an MCDA assay targeting the *tcdB* gene for detecting toxigenic *C. difficile* as a potential point-of-care testing method. The performance of the MCDA assay was assessed for detecting toxigenic *C. difficile* in clinical stool specimens and compared with real-time PCR and the VIDAS *C. difficile* toxin A & B assay (CDAB).

## MATERIALS AND METHODS

### MCDA primer design

The *C. difficile tcdB* gene (GenBank: KC292162.1) was used as the target for MCDA. MCDA primers were designed using Primer3 (v0.4.0) (https://bioinfo.ut.ee/primer3-0.4.0/), and the specificity of primers was verified using National Center for Biotechnology Information Primer BLAST and by alignment of different *tcdB* gene variants (24). Each primer set comprised 10 primers, including two cross-primers (CP1 and CP2), two displacement primers (F1 and F2), and six amplification primers (C1, C2, D1, D2, R1, and R2) designed according to the principle previously described (21).

### Clinical specimens, bacterial strains, and genomic DNA extraction

Stool specimens from patients suspected of CDI were consecutively collected from Zhejiang Provincial People’s Hospital from 1 to 31 October 2021. In accordance with the IDSA/SHEA guidelines, specimens were obtained from patients who had experienced ≥3 episodes of unexplained diarrhea within 24 h and had a history of antibiotic use (18). CDI was confirmed using the Xpert *C. difficile* assay following the manufacturer’s instructions (25). A sufficient volume of liquid, soft, or semisolid stool samples was tested using MCDA, real-time PCR, VIDAS CDAB, and cell cytotoxicity neutralization assay (CCNA), respectively. Genomic DNA from clinical stool samples was extracted using a Viral DNA Extraction Kit according to the manufacturer’s instructions (OriginGene Biotechnology Co., Ltd., Beijing, China). This study was approved by the Ethics Committee of the Hangzhou Medical College (LL2021-03). Written informed consent was waived due to the retrospective nature of this study.

The toxigenic *C. difficile* (*n* = 6), non-toxigenic *C. difficile* (*n* = 2), and diarrhea-associated strains (*n* = 27) used in this study are presented in Table S1. These strains were cultured according to the methods outlined in the Clinical Microbiology Procedures Handbook (26). Genomic DNA was extracted using the QIAamp DNA Mini Kit (QIAGEN, Inc., Valencia, CA, USA) according to the manufacturer’s instructions.

### MCDA reaction

MCDA reactions were carried out on a conventional real-time PCR instrument (Bioer LineGene 9600 Plus, Bioer Technology Co., Ltd., Hangzhou, China) using the WarmStart LAMP DNA Amplification Kit (New England Biolabs, Beijing, China). Genomic DNA extracted from the standard *C. difficile* strains (ATCC BAA-1870 and BAA-1801) was used as positive and negative controls, respectively.

Each MCDA reaction mixture contained 5 µL 2× WarmStart LAMP Master Mix, 0.2 µL 5× fluorescent dye, 1.2 µL MCDA primer mixture, 2.6 µL H_2_O, and 1 µL DNA template. Four sets of MCDA primers were tested under different temperatures ranging from 60 to 65°C, with 1°C increments to optimize the MCDA reaction conditions. MCDA amplification was monitored, with fluorescent measurements taken every minute. Positive, negative, and blank controls were included in each run.

### Real-time PCR for detection of toxigenic *C. difficile*

Real-time PCR reactions were carried out on the Bioer LineGene 9600 Plus instrument. The PCR primers and probe were designed to target the *tcdB* gene (GenBank: KC292162.1) using Primer Premier v3.0 (Applied Biosystems, MA, USA) (Table S2), and the specificity of primers was verified as described for MCDA above. The reaction was performed with the following cycling conditions: pre-denaturation at 95°C for 5 min, and then 40 cycles of 95°C for 15 s and 60°C for 45 s. Positive, negative, and blank controls were included in each run.

### VIDAS CDAB

The VIDAS CDAB assay was performed according to the manufacturer’s instructions (vidas_c._difficile_gdh_cdab_prn_18-0023-02_0.pdf.coredownload.pdf [biomerieux.com]) (27, 28). Well-mixed liquid stool (200 µL) was added to 1 mL of diluent and centrifuged for 5 min at 12,000 × *g*. The supernatant fluid (300 µL) was added to the sample wells for testing. Results were determined as positive, equivocal, or negative according to the intensity of the fluorescence.

### CCNA

The CCNA assay was performed as previously described (29). Briefly, 200 µL of well-mixed liquid stool was added to 1 mL of phosphate-buffered saline and filtered through a 0.22 µm microporous filter membrane (Merck KGaA, Darmstadt, Germany). Twenty microliters of the filtered sample was then added to a monolayer of both *C. difficile* toxin B neutralization antibodies (Diagnostic Hybrids, Inc., Athens, United States)-protected and -unprotected Vero cells (10^5^ cells/mL)-at 37°C containing 5% CO_2_ for 48 h. A stool sample was considered toxin positive when cell rounding was observed.

### Comparison of the limit of detection and speed between MCDA and real-time PCR

The limit of detection (LoD) and the speed of MCDA were compared to real-time PCR. Genomic DNA extracted from the standard strain (ATCC BAA-1870) was measured using a NanoDrop Lite Plus spectrophotometer (Thermo Fisher Scientific, Inc., Waltham, MA, USA) and serially diluted to 500, 50, and 5 pg and 500, 50, 25, 12.5, and 6.25 fg per μL. The *C. difficile* genomic DNA copy number for each dilution was calculated using an online copy number calculator (https://www.technologynetworks.com/tn/tools/copynumbercalculator) with the concentration of 25 fg calculated to be equivalent to 5 copies of *C. difficile* genomic DNA. All concentrations were independently tested six times in parallel by MCDA and real-time PCR. To compare the speed of real-time PCR with that of MCDA, the cycle threshold (Ct) was converted to time using the following equation as previously reported: Time = (Ct × 77.5 s) + 300 s (23). The detection time for real-time PCR was calculated based on the cycling conditions (60 s per cycle + an initial 300 s) and the ramp rate for Bioer LineGene 9600 Plus (Bioer Technology, Hangzhou, China) (17.5 s per cycle) according to the manufacturer’s technical information (https://www.biozym.com/media/pdf/77/52/9c/680000_LineGene96Plus.pdf).

### Data analysis

The CCNA was used as the reference standard for the detection of toxigenic *C. difficile*. Clinical sensitivity, specificity, positive predictive values (PPV), and negative predictive values (NPV) of MCDA were calculated and analyzed using the chi-squared test and compared to VIDAS CDAB and real-time PCR. The costs per test were approximately determined based on the prices of the reagents, which were purchased when the study was conducted. Analysis was carried out using SPSS version 23.0 (SPSS Inc., Chicago, IL, USA). Odds ratio (OR), 95% confidence interval (CI), and *P* values were calculated, and *P* values ≤ 0.05 were considered statistically significant.

## RESULTS

### Development and optimization of MCDA for detection of toxigenic *C. difficile*

**Fig 1:**
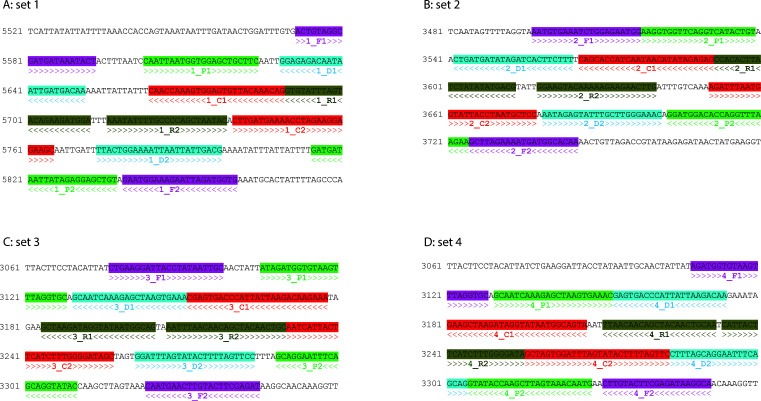
Locations of four MCDA primer sets designed in this study. A: Primer set 1. B: Primer set 2. C: Primer set 3. D: Primer set 4. Primers F1 and F2 were marked in purple, primers P1 and P2 were marked in green, primers D1 and D2 were marked in aqua, primers C1 and C2 were marked in red, and primers R1 and R2 were marked in olive.

**Fig 2:**
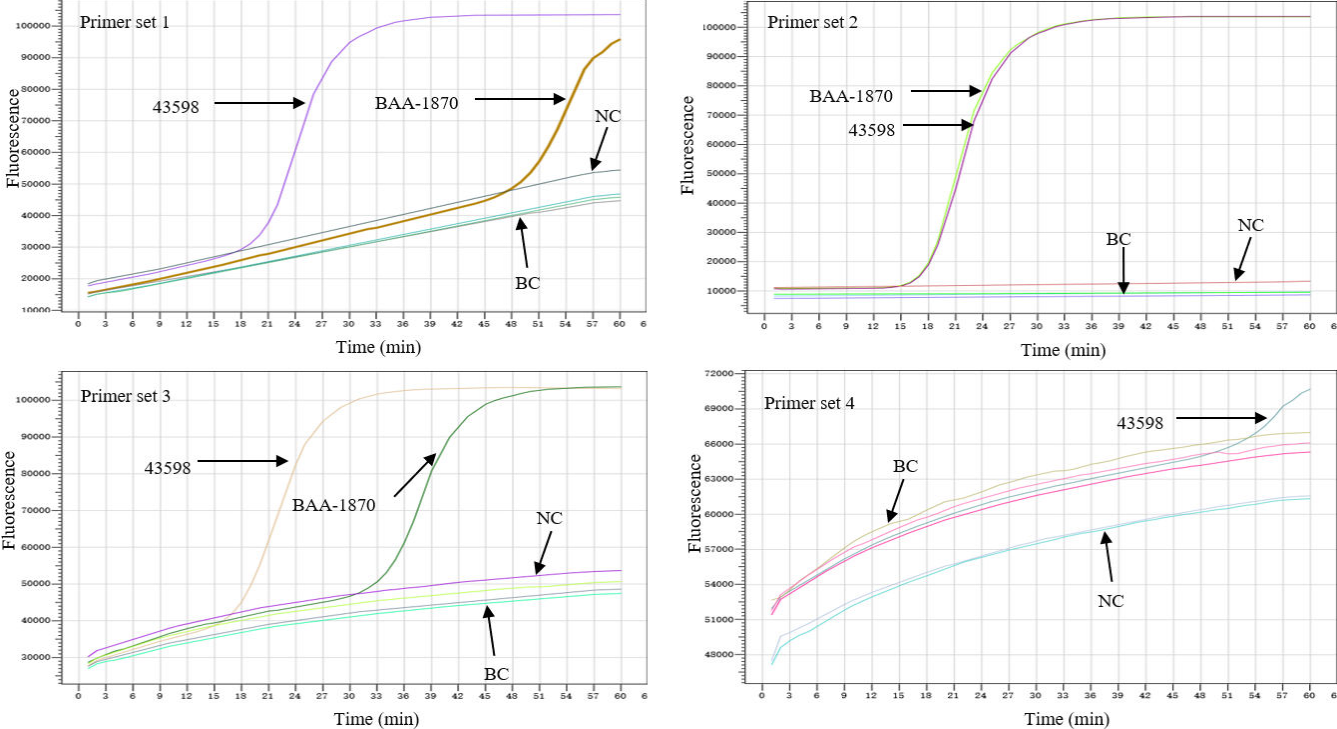
Initial evaluation of four MCDA primer sets. Two standard *C. difficile* strains (ATCC 43598 and BAA-1870) were used to evaluate the four MCDA primer sets. BC, blank control; NC, negative control.

**Fig 3:**
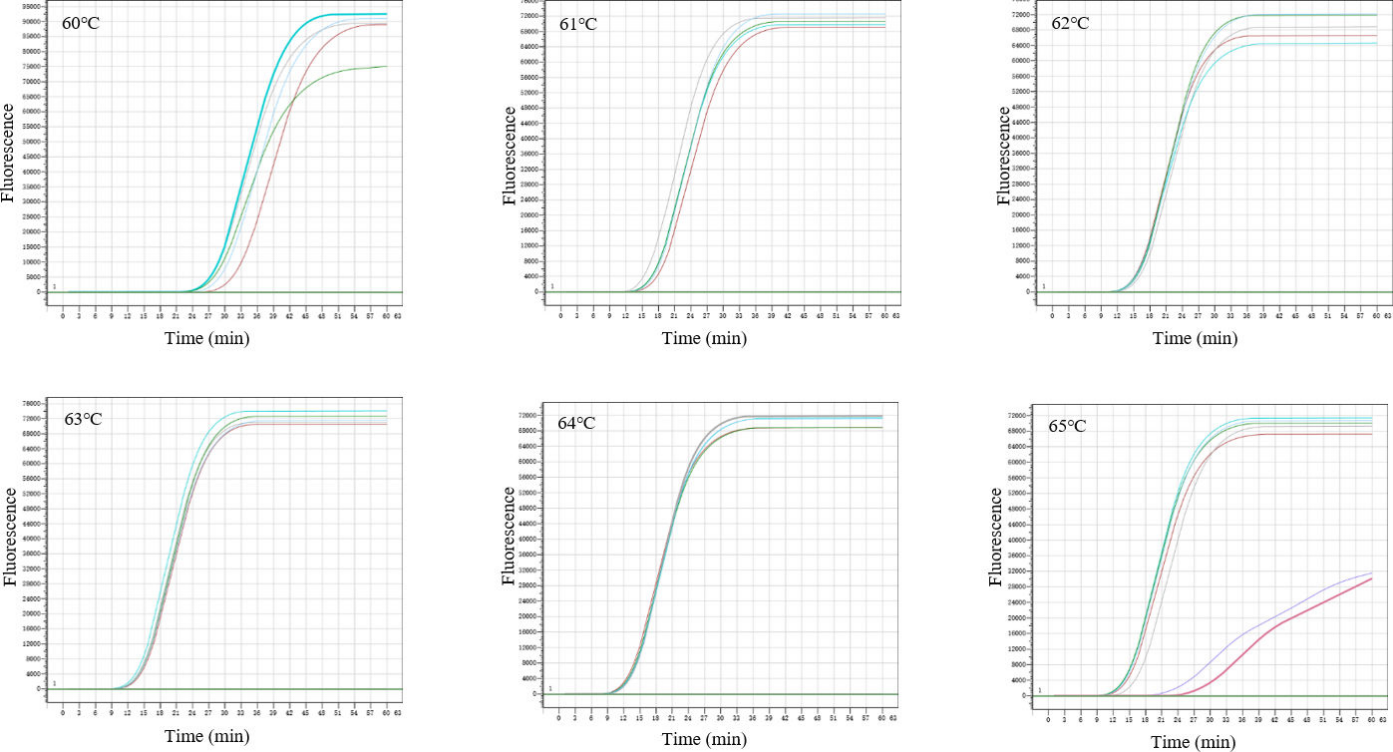
Optimization of MCDA with primer set 2 at different temperatures ranging from 60 to 65°C.

The sequences and locations of the four MCDA primer sets are shown in Table 1 and Fig. 1. When tested on two *C. difficile* strains (ATCC BAA-1870 and 43598), primer set 2 showed a robust performance, with a more stable, flatter fluorescence baseline and shorter detection time compared to the other three primer sets (Fig. 2). Primer set 2 was optimized for the *tcdB* gene amplification by testing temperatures ranging from 60 to 65°C, with six parallel runs per condition. The highest amplification efficiency and consistency were achieved at 63°C (Fig. 3).

**TABLE 1:** List of MCDA primers designed in this study

Primer name	Primers sequence 5′−3′	Length (bp)
MCDA primer set 1		
*tcdB*_I_F1	ACTGTAGGCGATGATAAATACT	22
*tcdB*_I_F2	CACCATCTAATTCTTTCCATTC	22
*tcdB*_I_P1	CAATTAATGGTGGAGCTGCTTC	22
*tcdB*_I_P2	ACAGCTCCTCTATAATTATCATC	23
*tcdB*_I_D1	TTGTCATCAATTATTGTCTCTCC	23
*tcdB*_I_D2	TTACTGGAAAATTAATTATTGACG	24
*tcdB*_I_C1	CTGTTTGTAACACTCCACTTTGGTTG	26
*tcdB*_I_C2	CTTGATGAAAACCTAGAAGGAGAAGC	26
*tcdB*_I_R1	TCCATCTTCTGTACTAAATACAC	23
*tcdB*_I_R2	AAATATTTTGCCCCAGCTAATAC	23
*tcdB*_I_CP1	CTGTTTGTAACACTCCACTTTGGTTGCAATTAATGGTGGAGCTGCTTC	48
*tcdB*_I_CP2	CTTGATGAAAACCTAGAAGGAGAAGCACAGCTCCTCTATAATTATCATC	49
MCDA primer set 2		
*tcdB*_II_F1	AATGTGAAATCTGGAGAATGG	21
*tcdB*_II_F2	TTGTGCCATCATTTTCTAAGC	21
*tcdB*_II_P1	AAGGTGGTTCAGGTCATACTGT	22
*tcdB*_II_P2	TTCTTAAACCTGGTGTCCATCC	22
*tcdB*_II_D1	AAAGAAGTGATCTATATCATCAG	23
*tcdB*_II_D2	AATAGAGTATTTGCTTGGGAAAC	23
*tcdB*_II_C1	CTCTCTATATGTTATTGATGGTGCTG	26
*tcdB*_II_C2	AGATTTAATGGTATTACCTAATGCTCC	27
*tcdB*_II_R1	CGTCATATATAGATAAGTGTGG	22
*tcdB*_II_R2	GGAAGTACAAAAAGAAGAACTTG	23
*tcdB*_II_CP1	CTCTCTATATGTTATTGATGGTGCTGAAGGTGGTTCAGGTCATACTGT	48
*tcdB*_II_CP2	AGATTTAATGGTATTACCTAATGCTCCTTCTTAAACCTGGTGTCCATCC	49
MCDA primer set 3		
*tcdB*_III_F1	CTGAAGGATTACCTATAATTGC	22
*tcdB*_III_F2	ATCTCGAAGTACAAGTTCATTG	22
*tcdB*_III_P1	ATAGATGGTGTAAGTTTAGGTGC	23
*tcdB*_III_P2	GTATACCTGCTGAAATTCCTGC	22
*tcdB*_III_D1	TTTCACTTAGCTCTTTGATTGC	22
*tcdB*_III_D2	GGATTTAGTATACTTTTAGTTCC	23
*tcdB*B_III_C1	TTTCTTGTCTTAATAATGGGTCACTCG	27
*tcdB*_III_C2	AATCATTACTTCATCTTTGGGGATAGC	27
*tcdB*_III_R1	CTGCCATTATACCTATCTTAGC	22
*tcdB*_III_R2	AATTTAACAACAGCTACAACTGC	23
*tcdB*_III_CP1	TTTCTTGTCTTAATAATGGGTCACTCGATAGATGGTGTAAGTTTAGGTGC	50
*tcdB*_III_CP2	AATCATTACTTCATCTTTGGGGATAGCGTATACCTGCTGAAATTCCTGC	49
MCDA primer set 4		
*tcdB*_IV_F1	AGATGGTGTAAGTTTAGGTGC	21
*tcdB*_IV_F2	TGCCTTATCTCGAAGTACAAG	21
*tcdB*_IV_P1	GCAATCAAAGAGCTAAGTGAAAC	23
*tcdB*_IV_P2	CATTGTTTACTAAGCTTGGTATAC	24
*tcdB*_IV_D1	TTGTCTTAATAATGGGTCACTC	22
*tcdB*_IV_D2	CTTTAGCAGGAATTTCAGCAG	21
*tcdB*_IV_C1	TACTGCCATTATACCTATCTTAGCTTC	27
*tcdB*_IV_C2	GCTAGTGGATTTAGTATACTTTTAGTTC	28
*tcdB*_IV_R1	TTGCAGTTGTAGCTGTTGTTAA	22
*tcdB*_IV_R2	CATTACTTCATCTTTGGGGATA	22
*tcdB*_IV_CP1	TACTGCCATTATACCTATCTTAGCTTCGCAATCAAAGAGCTAAGTGAAAC	50
*tcdB*_IV_CP2	GCTAGTGGATTTAGTATACTTTTAGTTCCATTGTTTACTAAGCTTGGTATAC	52

The final concentration for primer set 2 in the MCDA reaction mixture was 0.4 µM for F1 and F2, 2.4 µM for CP1 and CP2, 0.8 µM for C1 and C2, and 1.2 µM for R1, R2, D1, and D2. The MCDA reaction was performed at 63°C for 60 min, followed by a heating step at 95°C for 5 min to inactivate the amplification. Subsequently, the specificity of MCDA with primer set 2 was evaluated against six toxigenic *C. difficile* strains, two non-toxigenic *C. difficile* strains, and 27 diarrhea-associated strains (Table S1). Only the six toxigenic *C. difficile* strains produced positive MCDA reactions with a sharp S-shaped amplification curve, while none of the other strains produced positive results.

### Evaluation of the LoD and speed of MCDA

The LoD and speed of MCDA were compared with real-time PCR targeting the *tcdB* gene using serial dilutions of genomic DNA as templates. MCDA achieved an LoD of 12.5 fg (equivalent to 2.5 copies of genomic DNA) per reaction, matching that of real-time PCR (Table 2; Fig. S1). However, MCDA had faster detection times than real-time PCR across all genomic DNA concentrations tested. Notably, 500 pg of genomic DNA was detected in 5.7 ± 0.5 min (median: 5.64 min) by MCDA, which was nearly four times faster than the detection time for real-time PCR (23.97 ± 0.3 min, median: 24.03 min). MCDA detected 12.5 fg of genomic DNA with an average time of 21.35 ± 2.2 min (median: 21.30 min), approximately half the time required for real-time PCR (46.6 ± 0.95 min, median: 46.58 min). Based on their respective LoD values, positive amplification was confirmed by detecting amplification within a 23.55 min incubation time for MCDA and at a Ct value of approximately 32.95 (equivalent to about 47.55 min) for real-time PCR.

**TABLE 2:** Data on the LoD and speed of MCDA and real-time PCR

Result	Concentration of genomic DNA per reaction							
	500 pg	50 pg	5 pg	500 fg	50 fg	25 fg	12.5 fg	6.25 fg
Real-time PCR (no. positive/no. tested)	6/6	6/6	6/6	6/6	6/6	6/6	6/6	0/6
Ct								
Median	14.73	18.03	21.71	24.84	28.95	30.39	32.19	0
Range^*a*^	0.55 (14.37–14.92)	2.24 (16.57–18.81)	3.81 (20.12–23.93)	4.80 (23.07–27.87)	4.93 (26.91–31.84)	4.14 (28.9–33.04)	2.23 (31.03–33.26)	0
Reaction time (min)								
Median	24.03	28.29	33.04	37.08	42.39	44.25	46.58	0
Range	0.71 (23.56–24.27)	2.90 (26.40–29.30)	4.92 (30.99–35.91)	6.20 (34.80–41.00)	6.37 (39.76–46.13)	5.35 (42.33–47.68)	2.88 (45.08–47.96)	0
MCDA (no. positive/no. tested)	6/6	6/6	6/6	6/6	6/6	6/6	6/6	0/6
Reaction time (min)								
Median	5.64	7.11	9.30	10.53	15.65	18.36	21.30	0
Range	1.16 (5.19–6.35)	1.37 (6.31–7.68)	2.30 (7.93–10.23)	5.18 (9.57–14.75)	4.88 (14.41–19.29)	4.65 (18.06–22.71)	2.52 (19.88–22.40)	0

^*a*^ Range: the value is the maximum parameter subtracted from the minimum parameter.

### Comparative performance of MCDA, real-time PCR, VIDAS CDAB, and CCNA using clinical stool specimens

A total of 201 stool specimens from patients diagnosed with CDI via the Xpert *C. difficile* assay were tested in parallel using MCDA, real-time PCR, VIDAS CDAB, and CCNA. Of the 201 stool specimens, 58 were positive for toxigenic *C. difficile* using the Xpert *C. difficile* assay and classified as clinical CDI cases. The CCNA was used as the reference standard for evaluating the performance of MCDA. Sensitivities, specificities, PPVs, and NPVs of the three assays are summarized in Table 3. For sensitivity, MCDA achieved 93.1%, which was significantly higher than VIDAS CDAB (34.5%, *P* < 0.001, OR = 25.65, 95% [CI] = 5.04–130.61) but had no significant differences compared to real-time PCR (86.2%, *P* = 0.39, OR = 2.16, 95% [CI] = 0.36–12.84). For specificity, MCDA (92.4%) had significantly higher specificity than the real-time PCR (80.8%, *P* = 0.002, OR = 2.90, 95% [CI] = 1.47–5.74) but had significantly lower specificity than VIDAS CDAB (100%, *P* < 0.001, OR = 0.03, 95% [CI] = 0.00–0.58). Regarding PPV, MCDA (67.5%) outperformed real-time PCR (43.1%, *P* = 0.017, OR = 2.74, 95% [CI] = 1.18–6.36) but did not reach the 100% PPV observed for VIDAS CDAB (*P* < 0.001, OR = 0.10, 95% [CI] = 0.01–1.78). For NPV, MCDA (98.8%) was significantly superior to VIDAS CDAB (90.1%, *P* < 0.001, OR = 8.78, 95% [CI] = 2.01–38.31) and was also higher than the real-time PCR (97.2%), though there were no significant differences found (*P* = 0.34, OR = 2.29, 95% [CI] = 0.41–12.68). Notably, the average turnaround time for MCDA was 16.08 ± 4.62 min, which was significantly shorter than the turnaround time required for real-time PCR (39.82 ± 4.44 min) for the same samples (Table 4). The per-test cost was approximately $1.20 for MCDA compared to about $2.20 for real-time PCR based on reagent costs (data not shown). Overall, MCDA provided a balanced performance with robust sensitivity, specificity, PPV, and NPV, as well as the shortest test time among the three assays for detecting toxigenic *C. difficile*.

**TABLE 3:** Performance of MCDA in comparison to real-time PCR and VIDAS CDAB for detection of toxigenic *C. difficile* in stool samples

Test	No. of isolates with indicated result^*a*^				Sensitivity % (95% CI)	Specificity % (95% CI)	PPV % (95% CI)	NPV % (95% CI)
	S+ T+	S+ T−	S− T+	S− T−				
MCDA (reaction time < 23.55 min)	27	2	13	159	93.1 (83.9–100)	92.4 (88.5–96.3)	67.5 (53.0–82.0)	98.8 (97.0–100)
Real-time PCR (Ct < 32.95)	25	4	33	139	86.2 (69.4–94.5)	80.8 (74.3–86.0)	43.1 (31.2–55.9)	97.2 (93.0–98.9)
VIDAS CDAB	10	19	0	172	34.5 (19.9–52.7)	100 (97.8–100)	100 (72.3–100)	90.1 (85.0–93.5)

^*a*^ S: standard; T: test; +: positive; −: negative.

**TABLE 4:** Median, average, and ranges for real-time PCR Ct and MCDA reaction time using genomic DNA extracted from stool samples

Assay result	Ct value/detection time
Real time PCR (total^*a*^)	
Ct	
Median, (IQR)	28.95 (28.17, 29.77)
Average ± SD	28.34 ± 2.93
Reaction time (min)	
Median, (IQR)	42.39 (41.38, 43.45)
Average ± SD	41.61 ± 3.79
Real-time PCR (MCDA+^*b*^)	
Ct	
Median, (IQR)	28.49 (24.26, 29.40)
Average ± SD	26.95 ± 3.44
Reaction time (min)	
Median, (IQR)	41.79 (36.34, 42.98)
Average ± SD	39.82 ± 4.44
MCDA reaction time (total^*a*^) (min)	
Median, (IQR)	17.36 (12.86, 19.37)
Average ± SD	16.08 ± 4.62

^*a*^ Total: results from all the stool samples were positive for toxigenic *C. difficile*.

^*b*^ +: results from all the stool samples were positive for toxigenic *C. difficile* by MCDA.

## DISCUSSION

CDI, caused by toxigenic *C. difficile*, remains a significant healthcare challenge due to its potential for severe complications and high transmission rates (14). Rapid detection of toxigenic *C. difficile* is crucial for early intervention and epidemic control of CDI in primary healthcare facilities, especially in resource-poor settings. Although current quantitative PCR-based approaches have facilitated the CDI diagnosis and accelerated turnaround time compared to conventional assays, their application remains limited to laboratory settings due to the requirement for sophisticated instruments and specialized facilities (10, 16). Many isothermal nucleic acid amplification techniques with different mechanisms have been used to directly detect toxigenic *C. difficile* in stool samples (30), allowing rapid and easy-to-use operations for CDI diagnosis in various settings. Although they lack the ability to distinguish between CDC and CDI because of poor specificity (14), the IDSA/SHEA guidelines recommend molecular assays for timely diagnosis of patients with ≥3 episodes of unexplained diarrhea within 24 h and recent antibiotic use, especially for patients with severe clinical symptoms and when institutions have pre-agreed criteria for stool sample submission (18). Recent systematic review and meta-analysis also found that all-cause mortality was similar between patients that were molecular assay-positive/toxin-positive and patients that were molecular assay-positive/toxin-negative. This indicates that molecular assays with high sensitivities and high NPVs are meaningful for CDI diagnosis (17).

In this study, we developed and evaluated a novel MCDA assay as a promising alternative for directly detecting toxigenic *C. difficile* from clinical stool samples, offering rapid turnaround, user-friendly operation, and balanced performance. This study was also the first to benchmark the differences between MCDA and real-time PCR using the same reaction volume, machine, target gene, and clinical samples. Compared to real-time PCR, MCDA was performed at a constant temperature during the reaction stage without the need for temperature-controlling equipment (20). Additionally, MCDA demonstrated a LoD of 12.5 fg per reaction with a reaction time of 23.55 min, demonstrating a robust performance. These features make it a promising approach for the rapid detection of toxigenic *C. difficile* in clinical laboratories and point-of-care settings. Although MCDA could not bridge the CDI diagnostic gap for discriminating CDI from CDC, the higher NPV of MCDA may be used to rapidly exclude patients with non-toxigenic *C. difficile* for accurate CDI diagnosis.

TcdB is the most important virulence factor for CDI-related diseases (31). Our recent study has classified the *tcdB* gene into eight subtypes with sequence variations (24). After aligning all *tcdB* gene variants, MCDA primers were designed to minimize sequence variations. As shown in Fig. 1, a set of 10 primers with 10 binding sites correctly hybridizing to *tcdB* sequences provided a high degree of detection specificity, which was verified by testing a panel of diarrhea-associated bacteria, non-toxigenic *C. difficile*, and toxigenic *C. difficile* with different *tcdB* subtypes using MCDA. This demonstrated that MCDA using 10 primers recognizes more regions of the target sequence with higher specificity than TaqMan real-time PCR, which only uses two primers and one hybridization probe (32). Previous studies have also shown that MCDA achieves higher sensitivity than loop-mediated isothermal amplification (LAMP), which uses six primers (21, 23). However, the requirement for more primers may limit the broader application of MCDA. When designing MCDA primers, it is crucial to comprehensively analyze target sequences considering factors, such as complex secondary structure, GC content, and potential primer dimer formation. Therefore, not all target genes may be suitable for detection by MCDA, and different sets of MCDA primers should be designed and optimized as needed for specific detection assays.

MCDA also offered several other advantages over real-time PCR, which typically require complex thermal cycling protocols and longer processing times (21, 23). Furthermore, the price of reagents for each MCDA reaction was lower than for each real-time PCR reaction. By operating at a constant temperature, MCDA enables rapid amplification of target DNA sequences, potentially reducing diagnostic variability that may arise from insufficient temperature accuracy and uneven heat conduction in modules of real-time PCR instruments (20). Although the LoD of MCDA was the same as real-time PCR when both targeted the *tcdB* gene, MCDA achieved this sensitivity significantly faster, often saving approximately 20 min and requiring less than half the amplification time needed by real-time PCR. In clinical practice, PCR-based CDI testing is typically performed in batches of two to three runs daily, with each run requiring approximately 1–2 h (20). Although reducing the amplification time by less than half compared to real-time PCR might seem insignificant for the detection of toxigenic *C. difficile*, MCDA might deliver rapid results that address the urgent clinical needs of emergency or severe patients. Other studies have also shown that the LoD for MCDA was approximately 10-fold higher than those of other isothermal amplification approaches when detecting other pathogens (21, 23). Isothermal nucleic acid amplification approaches (33–35) have been used to detect *C. difficile*, targeting genes, such as *tcdA*, *tcdB*, binary toxin genes, and antibiotic resistance genes (34). However, our results indicate that the LoD of MCDA reported here was 40- and 47-fold more sensitive than that of other assays in previous studies (34, 36). Furthermore, LAMP reactions are usually conducted within 60 min to enhance their sensitivity and reduce cross-reactions (19, 36–38). MCDA thus showed superiority over other isothermal nucleic acid amplification methods as a potential point-of-care testing method, with better sensitivity and shorter detection time, as demonstrated previously for the detection of SARS-CoV-2 (23). These heightened sensitivity and efficiency are particularly valuable for identifying toxigenic *C. difficile* in outbreaks or high-risk settings, such as hospitals and long-term care facilities, contributing to reducing the risk of toxigenic *C. difficile* transmission.

The performance of MCDA was further evaluated on 201 clinical stool samples, demonstrating superior detection capability compared to real-time PCR and VIDAS CDAB. The overall positivity rates were 19.9% (40/201) for MCDA, 28.9% (58/201) for real-time PCR, and 5.0% (10/201) for VIDAS CDAB. The MCDA and the real-time PCR had significantly higher sensitivities and NPVs but lower specificities and PPVs than VIDAS CDAB, indicating that molecular assays may have poor capabilities to discriminate between CDI and CDC. However, all patients enrolled in this study met the IDSA/SHEA guidelines for CDI diagnosis, and stool samples from patients with unexplained diarrhea were also tested by the Xpert *C. difficile* assay. However, two CCNA-positive cases were missed by MCDA, and four were missed by real-time PCR. In addition, 13 and 33 samples showed discordant results between MCDA and real-time PCR and between real-time PCR and CCNA, respectively. Based on typical symptoms of CDI and positive results by the Xpert *C. difficile* assay, 33 real-time PCR-positive results were considered true clinical CDI cases, suggesting that the CCNA-negative results might be false-negative. These false negatives may be attributed to the limited sensitivity of CCNA, which could lead to an underestimation of the specificities of both MCDA and PCR (29). Furthermore, of these discordant cases, 20 samples were negative by MCDA but positive for *tcdB* by the real-time PCR and the Xpert *C. difficile* assays. These discrepancies may be attributed to the presence of inhibitors in stool samples, which may affect the MCDA reaction. As reported, substances, such as bile acids, polysaccharides, and lipids in stool (39, 40), as well as physicochemical properties like pH (41) and ion concentration (42), can affect the *Bst* polymerase activity. Thus, optimizing nucleic acid extraction to effectively remove these inhibitors will be essential to improving the performance of MCDA. To further evaluate the performance of our MCDA assay, we plan to isolate *C. difficile* from these stool samples and detect toxin genes for identification of toxigenic *C. difficile*. Overall, the MCDA with balanced performance and speed not only facilitated the rapid and reliable detection of toxigenic *C. difficile* but also excluded patients with non-toxigenic *C. difficile*, thereby minimizing CDI transmission and unnecessary clinical treatment across various settings.

As described, molecular methods, including PCR- and isothermal nucleic acid amplification-based assays used as stand-alone tests, have poor specificities for discrimination between CDI and CDC (43). In current CDI diagnosis guidelines, two-step algorithms based on sensitive screening assays are often recommended to identify toxigenic *C. difficile* and achieve optimal diagnostic accuracy while reducing inappropriate CDI therapy (17). The first tests performed are usually molecular assays or a GDH test, as they have high sensitivities and high NPVs. For positive results, a follow-up test using the toxin A/B EIA is conducted to confirm toxigenic *C. difficile*. However, molecular assays alone can be used for CDI diagnosis if appropriate stool sample selection is guaranteed by clinical laboratories as mentioned in the IDSA/SHEA guidelines (18). Therefore, the MCDA reported here can be a promising alternative as a single test with high sensitivity and specificity, high NPV, rapid turnaround time, and low cost for detection of toxigenic *C. difficile*, although it does not discriminate between CDI and CDC. Furthermore, MCDA may also be used as the initial screening for the preliminary detection of toxigenic *C. difficile* in combination with highly specific confirmation tests to enhance the overall accuracy of CDI diagnosis in a two-step algorithm. Beyond its diagnostic capabilities, practical advantages of MCDA make it well-suited for diverse healthcare settings, including resource-limited environments. Its isothermal nature simplifies operational requirements, potentially lowering the barrier to adoption in settings where sophisticated laboratory infrastructure is difficult to establish or access.

There are some limitations to our study. First, all patients with CDI were diagnosed as positive by the Xpert *C. difficile* assay. However, detailed clinical information, including clinical symptoms and efficacy of antibiotic treatments, was not collected, as written informed consent was waived in this study. Moreover, the performance of MCDA was not compared to the GDH test, which has high sensitivity and a favorable NPV. Therefore, we aim to start another prospective cohort study to further evaluate the performance of MCDA for CDI diagnosis in comparison to a two-step GDH-toxin test, during which detailed clinical data would be collected to more accurately distinguish CDI from CDC. Second, the discrepant results obtained from the three different assays were not analyzed further in this study. The performance of MCDA should be confirmed by isolating *C. difficile* from stool samples to identify toxin genes and using a digital PCR assay for absolute quantitative analysis of these samples. Finally, the MCDA assay reported here was conducted on a conventional real-time PCR instrument in our laboratory. Although real-time PCR instruments are used routinely in clinical laboratories, the development of a compact isothermal amplification device integrated with lateral flow immunochromatographic strips for visual detection will further enhance portability, remove the need for large-scale instruments, reduce turnaround time, and meet point-of-care testing needs, particularly in community or resource-limited settings.

In conclusion, the MCDA assay developed in this study provides a rapid and preliminary screening tool for detecting toxigenic *C. difficile*, effectively excluding patients with non-toxigenic *C. difficile*, and for the timely prevention of CDI transmission. Its rapid detection capability, along with balanced sensitivity and specificity, makes it a promising screening alternative either alone or combined with other highly specific confirmatory tests for point-of-care testing in clinical diagnostics and infection control in clinical laboratories, primary healthcare facilities, and most especially resource-poor settings. Further studies should be conducted to evaluate its performance across larger patient cohorts and diverse epidemiological settings in comparison to a GDH test combined with toxin tests. The isolation of *C. difficile* for identification of toxin genes will also further validate the reliability, robustness, and suitable application areas.

## SUPPLEMENTAL MATERIAL

Figure S1 and Tables S1 and S2 spectrum.03030-24-s0001.pdf Fig. S1: LoDs achieved by MCDA and real-time PCR. Table S1: Bacterial strains used in this study. Table S2: Real-time PCR primers and probe designed in this study.

ASM does not own the copyrights to Supplemental Material that may be linked to, or accessed through, an article. The authors have granted ASM a non-exclusive, world-wide license to publish the Supplemental Material files. Please contact the corresponding author directly for reuse.
