# Supplementary material for: Multiple cross displacement amplification (MCDA) for rapid detection of toxigenic Clostridioides difficile as a potential point-of-care testing
Source: Microbiol Spectr. 2025 Jul 22;13(9):e03030-24. doi: 10.1128/spectrum.03030-24 (PMC12403592; doi:10.1128/spectrum.03030-24)
Supplement: Figure S1 and Tables S1 and S2 — Fig. S1: LoDs achieved by MCDA and real-time PCR. Table S1: Bacterial strains used in this study. Table S2: Real-time PCR primers and probe designed in this study. [file spectrum.03030-24-s0001.pdf]

Supplementary Figure Legends

Fig. S1. The LoDs achieved by MCDA and real-time PCR.

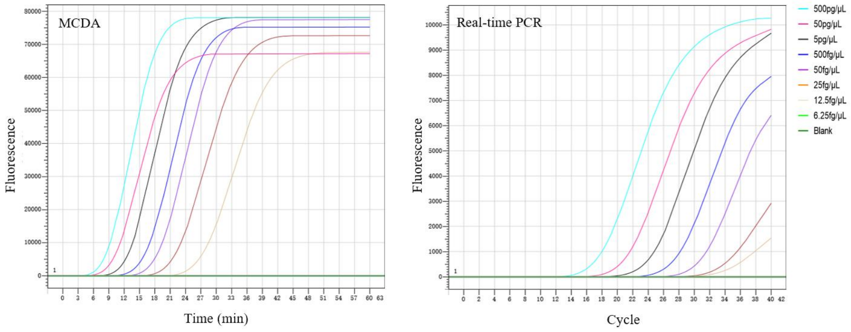

Fig. S1

Table S1. Bacterial strains used in this study

Bacterial strain	ATCC number
Listeria monocytogenes	54004
Listeria innoch	33090
Listeria ovis	19119
Listeria cerevisiae	35967
Listeria gasseri	25401
Staphylococcus aureus	26001
Citrobacter	48068
Streptococcus faecalis	32219
E. coli O157:H7	44752
Salmonella Enteritidis	50041
Salmonella typhi	50096
Salmonella typhimurium	14028
Salmonella	50001
Salmonella Paratyphi A	12176
Salmonella Paratyphi B	10719
Salmonella Paratyphi C	13428
Serratia marcescens	CMCC41002*
Klebsiella pneumoniae	CMCC46114
Proteus mirabilis	49027
Morganella morganii	CMCC49087
Bacillus cereus	63301
Vibrio cholerae	16025
Vibrio vulnificus	27562
Campylobacter jejuni	33560
Yersinia enterocolitica	52207
Shigella	51081
Vibrio parahaemolyticus	20502
Non-toxigenic *C. difficile*	BAA-1801, 700057
Toxigenic *C. difficile*	43255, BAA-1870, BAA-1875, BAA-1882, 43598, 9689

*: Three strains were obtained from the National Centre for Medical Culture Collections (CMCC) (www.cmccb.org.cn).

Table S2. The real-time PCR primers and probe designed in this study

Primer/probe name	Sequences 5'-3'	Product (bp)
tcdB-F	GATAATATTTACGGACAAGCAGTTGACT	
tcdB-R	AGTCTCAATTGTATAGGTTTCTCCAAAA	93
tcdB-Probe	FAM-ACCCGTTTAGTTACAGTTG-BHQ1	
